# PERIOPERATIVE NUTRITIONAL SUPPORT IN GASTROINTESTINAL SURGERY - WHY IS IT ESSENTIAL?

**DOI:** 10.1590/S0004-2803.24612024-94

**Published:** 2025-06-16

**Authors:** Daniéla Oliveira MAGRO, Amanda Pereira LIMA, Cláudio Saddy Rodrigues COY, Paulo Gustavo KOTZE

**Affiliations:** 1 Faculdade de Ciências Médicas da Universidade Estadual de Campinas, Departamento de Cirurgia, Campinas, SP, Brasil.; 2 Pontifícia Universidade Católica do Paraná, Programa de Pós-Graduação em Ciências da Saúde, Curitiba, SP, Brasil.

**Keywords:** ERAS protocol, immunonutrition, gastrointestinal surgery, malnutrition, obesity, Protocolo ERAS, imunonutrição, cirurgia gastrointestinal, desnutrição, obesidade

## Abstract

Background:

Malnutrition, sarcopenia, and obesity can negatively impact the course of gastrointestinal surgery, increasing the risk of postoperative complications such as anastomotic dehiscence, reoperations, increased mortality and morbidity, and prolonged hospitalizations, leading to higher healthcare costs. Weight loss greater than 10% in the previous six months is a prognostic indicator of mortality in gastrointestinal surgeries and one of the few modifiable variables. Preoperative malnutrition prevalence ranges from 17% to 20%, increasing the risk of infectious complications, especially in malignant diseases. Obesity, i.e., body mass index (BMI) ≥30.0 kg/m^2^, also impairs the clinical course, contributing to postoperative complications and hospital mortality. Enhanced recovery protocols, like ERAS, are becoming standard practice, with preoperative nutritional interventions crucial for improving surgical outcomes. However, there is no consensus on the ideal preoperative dietary intervention, but regardless of nutritional status, all individuals are eligible for preoperative screening. The American Gastroenterological Association (AGA) proposes to assess malnutrition using signs and symptoms, including unintentional weight loss, edema, loss of fat and body muscle mass, and fluid retention, in addition to BMI ≤18.5 kg/m^2^. In the case of malnutrition, the use of oral supplementation, enteral nutrition (EN), or parenteral nutrition (PN) is recommended, even if there is a need to postpone surgery. This article discusses the importance of nutritional status screening and perioperative nutritional support, emphasizing the need for a comprehensive approach to improve patients’ quality of life and reduce postoperative complications.

## HIGHLIGHTS

• Preoperative nutritional screening is crucial, with tools like the NRS recommended to identify at-risk patients.

• Preoperative nutritional support, including immunonutrition, has improved outcomes, particularly in malnourished patients.

• ERAS protocol incorporating nutritional support is becoming standard practice in managing patients undergoing gastrointestinal surgery.

• Postoperative nutritional support should be initiated immediately to minimize complications and promote recovery.

## RESUMO

**Contexto:**

Tanto a desnutrição, como a sarcopenia e a obesidade podem impactar negativamente o curso da cirurgia gastrointestinal, aumentando o risco de complicações pós-operatórias, como deiscência de anastomose, reoperações, aumento da mortalidade e morbidade, e hospitalizações prolongadas, resultando em custos elevados para o sistema de saúde. A perda de peso superior a 10% nos últimos 6 meses é um dos indicadores prognósticos de mortalidade em cirurgias gastrointestinais e uma das poucas variáveis modificáveis. A prevalência de desnutrição no pré-operatório varia de 17% a 20%, aumentando o risco de complicações infecciosas, especialmente em doenças malignas. A obesidade, ou seja, índice de massa corporal (IMC) ≥30.0 kg/m^2^, também prejudica o curso clínico, contribuindo para complicações pós-operatórias e mortalidade hospitalar. Protocolos de recuperação aprimorada, como o *Enhanced Recovery After Surgery (ERAS)* estão se tornando práticas padrão, incluindo intervenções nutricionais pré-operatórias, essenciais para melhorar os desfechos cirúrgicos. No entanto, não há consenso sobre a intervenção nutricional ideal no pré-operatório, porém, independente do estado nutricional, todos os indivíduos são elegíveis a triagem pré-operatória. A Associação Americana de Gastroenterologia propõe avaliar a desnutrição usando sinais e sintomas, incluindo a perda de peso não intencional, edema, perda de gordura e massa muscular corporal e retenção de líquidos, além do IMC ≤18.5 kg/m^2^. No caso de desnutrição, recomenda-se o uso de suplementação oral ou nutrição enteral (NE) ou nutrição parenteral (NP), mesmo que haja necessidade de adiar a cirurgia. Este artigo discute a importância da triagem do estado nutricional e do suporte nutricional perioperatório, destacando a necessidade de uma abordagem abrangente para melhorar a qualidade de vida dos pacientes e reduzir complicações pós-operatórias.

## INTRODUCTION

Malnutrition, sarcopenia (loss of muscle mass)^1^, and obesity can negatively impact the course of gastrointestinal surgery, leading to increased risks of postoperative complications, such as anastomotic dehiscence, reoperations, increased mortality (relative risk 1.6-1.9)^2^ and morbidity, and prolonged hospitalizations (1.5-1.7 times longer)^2^, which have been shown to increase healthcare costs^3^ ^,^ ^4^. However, we can work towards improving patient outcomes by understanding these risks and implementing appropriate measures.

Weight loss greater than 10% in the previous six months was one of sixteen prognostic indicators of mortality in people undergoing gastrointestinal surgery and is one of the few variables considered adjustable^3^. 

Malnutrition preoperative prevalence occurs in 17% to 20%^3^. It increases the risk of infectious complications after gastrointestinal surgery, mainly in malignant diseases with esophagus, stomach, colorectal, pancreatic, and liver cancers, such as inflammatory bowel diseases^4^. There is variation in reported data about malnutrition in gastrointestinal surgical procedures, possibly due to different techniques used to measure nutritional screening for the risk of malnutrition^3^.

The severity of malnutrition is influenced by the duration and extent of the disease, particularly the inflammatory response, which increases metabolic activity, leading to catabolism and sarcopenia and compromising immunity^2^. The prevalence of sarcopenia in cancer patients with chemotherapy can be up to 83%^1^.

Vitamin and mineral deficiencies are common in malnutrition. They generally reflect losses due to bleeding (e.g., iron deficiency) and diarrhea (e.g., hypomagnesemia), reduced absorption due to intestinal resections (vitamin B12, vitamin D), or more diffuse malabsorption (most micronutrients), loss of appetite, and hypercatabolic^5^. The most common micronutrient deficiencies are iron, calcium, selenium, zinc, magnesium, vitamin B12, folic acid, and fat-soluble vitamins such as A, D, and K^4^. 

Obesity (body mass index ≥30 kg/m^2^) also harms the patient’s clinical journey, leading to postoperative complications, such as infections and anastomotic leakage, impaired healing thromboembolic complications, prolonged hospital stay, and rehabilitation^6^. Sarcopenic obesity is often masked in overweight or obese patients and contributes to hospital mortality in 7% of obese patients^1^. 

Studies that addressed changes in perioperative management for patients undergoing gastrointestinal surgery emerged in the 1990s^7^. Enhanced recovery after surgery (ERAS) is becoming standard practice in managing patients undergoing gastrointestinal surgery^3^. These changes, including oral supplementation or enteral or parenteral nutritional support, shortening preoperative fasting, and using anesthetic techniques for pain management, refeeding, and early mobilization, have shown promising benefits in surgical recovery^3^ ^,^ ^4^ ^,^ ^8^. Adopting the ERAS protocol is a significant advancement in the field, providing a comprehensive approach to patient care.

However, no consensus exists regarding nutritional intervention in the preoperative for patients admitted for elective gastrointestinal surgery^3^.

### Screening of nutritional status

It is estimated that only 40% of malnourished patients are treated perioperatively^2^ ^,^ ^9^. Patients undergoing gastrointestinal surgery must be available for preoperative nutritional status screening. Nutritional assessment requires a multifaceted approach, encompassing anthropometric measurements and evaluation of body composition (bioelectrical impedance analysis (BIA), dual-energy X-ray, absorptiometry (DXA), computed tomography (CT), and magnetic resonance imaging (MRI))^10^, dietary intakes, biomarkers of nutritional status, such as albumin and micronutrients, clinical examinations, and environmental and socioeconomic factors^4^. 

More than 70 nutritional screening tools, from the simplest to the most complex methods, have been proposed for assessing the nutritional status of malnourished patients^2^. Among these tools, we highlighted the Nutrition Risk Score (NRS), according to Kondrup^11^, a technique recommended by the European Society for Enteral and arenteral Nutrition (ESPEN), which has been well-validated for surgical patients^1^. The NRS variables include unwanted weight loss (amount and time), BMI for adults, appetite, chewing and swallowing ability, gastrointestinal symptoms (vomiting and diarrhea), factors related to the underlying disease, stress, and the patient’s clinical conditions. Severe nutritional risk, including NRS score ≥3^11^. The (AGA) proposes to assess malnutrition using signs and symptoms, including unintentional weight loss, edema, loss of fat and body muscle mass^10^. 

Malnutrition and poor nutritional intake after surgery can contribute to worse psychological health and negatively impact the quality of life of patients^3^.

### Preoperative nutritional support

**FIGURE 1:**
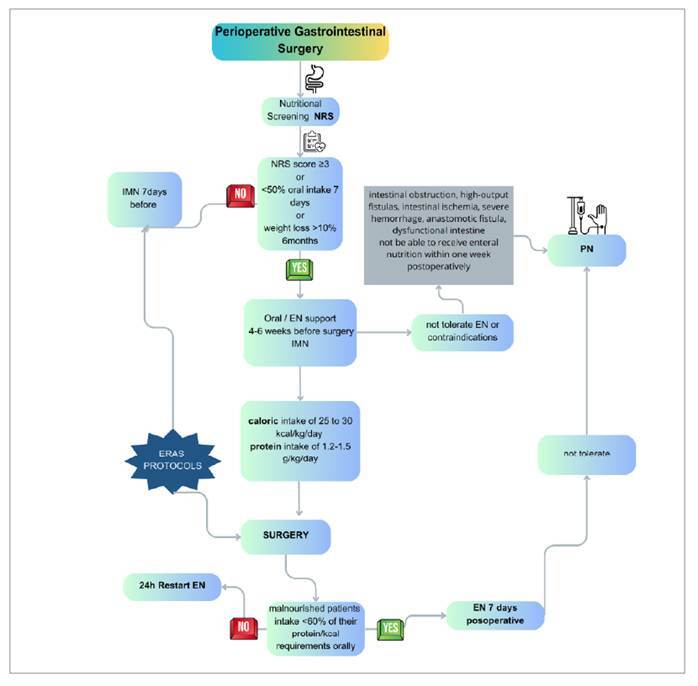
Perioperative nutritional support in gastrointestinal surgery. NRS is conducted to identify patients at risk of malnutrition. Those identified as high-risk receive specific nutritional support, including IMN, to optimize the immune system and promote healing. The ERAS protocol is implemented to improve postoperative recovery, incorporating practices such as reduced preoperative fasting and early mobilization. Depending on the patient’s nutritional status and oral intake capacity, EN is preferred, but PN is indicated when EN is not feasible.

NRS: nutritional risk screening; IMN: immunonutrition; ERAS: enhanced recovery after surgery; EN: enteral nutrition; PN: parenteral nutrition. Adapted from Wobith M, Weimann A, 2021^1^.

The ESPEN has recommended assessing nutrition status during preoperative (Figure 1). This period is intended to correct energy deficits and improve nutritional status with adequate protein supplementation^3^ and immunonutrition^1^. 

Serum proteins, vitamins A, C, and B complex, amino acids (arginine, glutamine, and proline), iron, zinc, and selenium benefit wound healing and optimize the immune system. Protein plays a critical role in the healing process^12^. Protein demands are increased during surgery. Metabolic stress causes an increase in protein breakdown and utilization without increasing protein synthesis^2^. Minor surgeries have shown decreased prealbumin levels postoperatively in patients receiving only oral nutrition^12^.

The immunomodulatory diet has been proposed as a risk-reduction strategy in surgical patients for over 25 years. Immunonutrition (IMN) describes the enteral/oral administration of substrates (arginine, fatty acids, omega 3, nucleotides, glutamine, and antioxidants) with immunomodulatory function. The most significant evidence for IMN is malignant pathologies, with decreased postoperative complications, including lower C-reactive protein (CRP) levels and reduced pain^1^ ^,^ ^13^ ^,^ ^14^. Arginine can be used in collagen synthesis and is essential in cell proliferation and differentiation. It is a precursor to nitric oxide, and proline is one of the main extracellular components of connective tissue. It acts in antioxidant reactions and immunological responses and is essential for anastomosis, healing, and improving pressure ulcers^13^.

Perioperative nutritional support therapy is indicated in patients with malnutrition and those at nutritional risk or who have low oral intake and who cannot maintain above 50% of the recommended intake for more than seven days^1^. Oral or enteral nutritional support should be preferred exclusively for 4 to 6 weeks before elective surgery. Its advantages include lower cost, avoiding intravenous access and associated complications, maintaining epithelial integrity and immunomodulation, effects on the microbiota, and providing clinical benefits for the attenuation of weight loss^1^. The ESPEN guidelines recommend a caloric intake of 25 to 30 kcal/kg/day and a protein intake of 1.2-1.5 g/kg/day.

Enteral nutrition (EN) can be administered orally or via tube, with similar effects. In patients who can ingest food orally but do not fully meet their caloric needs (caloric supplement <50% of calories from formula or partial enteral nutrition - 50-60% of calories from formula)^5^, EN can be used to slowly infuse nutrients in a controlled manner and reduce intolerance associated with the food bolus. It should preferably be administered at night so as not to impair spontaneous oral feeding^15^.

Currently, EN is preferable to parenteral nutrition (PN) due to the lower incidence of complications and cost^16^. In addition, EN allows nutrients in the intestinal lumen, which acts as a trophic factor, preventing bacterial translocation and preserving gastrointestinal function^15^. The largest retrospective cohorts suggest that patients who proceed directly to surgery are five times more likely to develop intra-abdominal septic disease than those who receive exclusive enteral nutrition (EEN) (≥90% of calories from formula)^17^.

Indications for PN are reserved for patients who do not tolerate EN, for cases of intestinal obstruction, high-output fistulas, intestinal ischemia, severe hemorrhage, anastomotic fistula, dysfunctional intestine, or for patients who will not be able to receive enteral nutrition within 1 week postoperatively. Complications related to PN are divided into mechanical (related to the passage or maintenance of the catheter), septic (catheter-associated infections), and metabolic (hydroelectrolytic disorders, refeeding syndrome, cholestatic syndrome, and bone mineral disease)^3^ ^,^ ^8^ ^,^ ^16^.

From a practical point of view, there is no clear evidence for the time frame before surgery^5^. The important thing is to correct the patient’s nutritional status before surgery, and preoperative nutrition supplementation is found to reduce postoperative complications. EN is superior to the standard of care without nutrition support^3^. Consequently, we could direct preoperative nutritional support as follows:

- NE for 4 to 6 weeks before elective surgery with a caloric supplement, partial enteral nutrition, or NEE^1^.
- An immunomodulatory diet could be indicated for 7 days before elective surgery^1^ ^,^ ^18^. ERAS Protocol (abbreviated fasting (up to 3 h) and ingesting a carbohydrate-enriched solution)^9^.
- PN should only be used when adequate EN is not feasible^16^.

### Postoperative nutritional support

Postoperative enteral nutrition should, whenever possible, be restarted within the first 24 hours in cases of malnourished patients and in those in whom patients do not meet adequate nutritional goals (intake of at least 60% of their protein/kcal requirements orally) since, when performed early, morbidity and mortality rates are reduced^2^ ^,^ ^15^. There is still no consensus on the ideal time for nutritional support in the postoperative period; however, studies suggest that nutritional therapy, when administered between 3 and 10 days (7 days being the most common period), is associated with a significant benefit in the recovery of the nutritional status and quality of life of patients with malnutrition^15^. The postoperative nutritional support could be:

- An immunomodulatory diet could be indicated for 7 days after elective surgery^1^.
- A PN could be indicated for those who cannot receive enteral nutrition within 1 week postoperatively^16^.

## CONCLUSION

Optimizing nutritional status before and after gastrointestinal surgery is essential for improving patient outcomes. Current guidelines strongly recommend nutritional screening, preoperative support when indicated, and the implementation of ERAS protocols to ensure best-practice care. However, future studies on the best practices for nutritional interventions in the pre- and postoperative periods and greater adherence to perioperative nutritional protocols are needed. 
